# Reducing Sedentary Time for Obese Older Adults: Protocol for a Randomized Controlled Trial

**DOI:** 10.2196/resprot.8883

**Published:** 2018-02-12

**Authors:** Dori E Rosenberg, Amy K Lee, Melissa Anderson, Anne Renz, Theresa E Matson, Jacqueline Kerr, David Arterburn, Jennifer B McClure

**Affiliations:** ^1^ Kaiser Permanente Washington Health Research Institute Seattle, WA United States; ^2^ University of California San Diego San Diego, CA United States

**Keywords:** Sedentary lifestyle, exercise, aging, chronic conditions, medical informatics

## Abstract

**Background:**

Older adults have high rates of obesity and are prone to chronic health conditions. These conditions are in part due to high rates of sedentary time (ST). As such, reducing ST could be an innovative strategy for improving health outcomes among obese older adults. To test this theory, we developed a novel, technology-enhanced intervention to reduce sitting time (I-STAND) and pilot tested it to assess the feasibility, acceptability, and preliminary effects of the intervention on ST and biometric outcomes.

**Objective:**

The current paper aims to describe the rationale, design, and methods of the I-STAND sitting reduction pilot trial.

**Methods:**

Older adults with obesity (n=60) were recruited from a large health care system and randomized to receive I-STAND or a healthy living intervention. I-STAND combined personal coaching with a technology-enhanced intervention (Jawbone UP band) to cue breaks from sitting. Participants completed self-report and biometric assessments at baseline and 3 months. Additional qualitative results were collected from a subset of I-STAND participants (n=22) to further inform the feasibility and acceptability of the interventions. The primary outcome was total hours of daily sitting time measured by the activPAL device. Secondary outcomes included sit-to-stand transitions, bouts of sitting longer than 30 minutes, physical function, blood pressure, fasting glucose, cholesterol, and depressive symptoms.

**Results:**

Study enrollment has ended and data processing is underway.

**Conclusions:**

Data from randomized trials on sitting reduction are needed to inform novel approaches to health promotion among older adults with obesity. Our trial will help fill this gap. The methods used in our study can guide future research on using technology-based devices to assess or prompt sedentary behavior reduction, or those interested in behavioral interventions targeting obese older adults with novel approaches

**Trial Registration:**

ClinicalTrials.gov: NCT02692560; https://clinicaltrials.gov/ct2/show/NCT02692560 (Archived by WebCite at http://www.webcitation.org/6wppLTWAl)

## Introduction

Currently, one-third of adults over the age of 60 are affected by obesity [1], and rates are projected to double between 2000 and 2030 among those 65 and older [2]. The consequences of obesity in older adulthood include poor physical function, increased disability, elevated risk of morbidity (arthritis, diabetes) and mortality, decreased quality of life, and higher healthcare costs [3-5]. Novel behavioral interventions are needed to reduce these problems and those associated with other aging-related chronic conditions.

Increased physical activity, particularly at moderate-to-vigorous levels, promotes healthy weight and has a plethora of positive health effects [6,7]. Yet, of all age groups, older adults are the least likely to meet physical activity guidelines (2.4% of adults aged 65 and older by objective measures) [8]. Many older adults may be unable or unwilling to increase their level of physical activity, and reducing sedentary behavior could be a more feasible goal.

Sedentary behavior has been defined as “any waking behavior characterized by an energy expenditure <1.5 metabolic equivalents while in a sitting or reclining posture“ [9-11]. Common sedentary activities among older adults include watching television, doing seated activities (eg, knitting, reading, attending seated events), and riding in an automobile [10,12,13]. Lab studies suggest that sitting adversely impacts cardiometabolic health markers such as triglycerides, glucose, and insulin sensitivity [14]. Sedentary time (ST) is high among older adults at 8 to 10 hours per day or 65% to 70% of waking hours [15-17]. Older adults with obesity tend to have an even higher ST at 10 to 11 hours per day [16,18].

Reducing sitting behaviors among older adults with obesity could promote health benefits. Randomized trials of sitting reduction have been promising in adult populations, particularly those based in the workplace [19,20]. However, data from randomized studies are lacking among older populations. The majority of studies are small pretest posttest studies. These studies indicate preliminary feasibility of reducing ST, achieving around 30 to 50 minute reductions in sitting time per day [21-27]. Only one of these studies, conducted by our team, focused on older adults with obesity and found reductions in sitting were feasible and consistent with reductions in non-obese older adults [22]. Building on our preliminary work, we developed the I-STAND intervention which combines our prior cognitive behavioral intervention with new elements such as prompts to stand delivered by a wrist-worn activity sensor (Jawbone UP band) and biomarker assessments. To determine the feasibility, acceptability, and preliminary behavioral and health effects of the I-STAND intervention, we conducted a randomized controlled pilot trial. The current paper details the rationale, design, and methods of this trial.

## Methods

### Trial Design

A 12-week single-blind, randomized two-arm trial design was employed to evaluate the efficacy of the I-STAND intervention for decreasing sitting time compared to a healthy living control group. Enrollment began in February 2016 and data collection finished in February 2017.

### Setting

The study is being conducted by the Kaiser Permanente Washington Health Research Institute (formerly, Group Health Research Institute). All activities were reviewed and approved by the Kaiser Permanente Washington (KPWA) Institutional Review Board.

### Recruitment

Potential participants were identified using electronic health records from members of Kaiser Permanente Washington. Participants were limited to members whose primary care clinics were located in King County, WA to facilitate in-person appointments. Individuals were deemed potentially eligible if their: electronic medical records indicated they were aged 60-89, body mass index was ≥30 (to select for a group at risk for chronic conditions who may benefit the most from ST reduction), and enrollment in the health plan was continuous for the prior 12 months. Individuals were excluded if they resided in long-term care or a skilled nursing facility in the prior 12 months, had a new cancer or heart failure diagnosis, or had a new diagnosis of dementia or serious mental health disorder.

Study invitation letters were mailed to a random selection of potentially eligible individuals who met the criteria above. Those who were interested in learning more were asked to call study staff for more information. Up to three mailings were sent to potential participants if they did not respond to the initial invitation or opt out of further contact. Interested responders were screened for eligibility by phone. Additional eligibility requirements were: self-report of sitting ≥ 7 hours per day, able to stand, and able to walk one block with or without an assistive device.

### Contacts and Procedures

Persons screened as potentially eligible by phone provided oral consent to participate and were scheduled for an in-person appointment. They were then mailed an activPAL device. activPAL is currently considered the most valid and objective measure of sitting time [28]. This small lightweight device was worn on the front-middle part of the thigh with a waterproof dressing. Participants were provided with clear instructions and photos showing them how to adhere the device to their leg. The device was worn on the leg 24 hours a day to assess active and sitting time. Participants wore the device for at least 7 days prior to coming to an in-person baseline assessment. Participants completed a log to record their sleeping hours.

At the in-person baseline visit, participants met with a study staff member who collected written informed consent, downloaded their activPAL data, and collected other baseline assessment data (including a questionnaire, biometric assessments, and a fasting blood draw). A separate study health coach then randomized individuals and met with them to inform participants of their randomization group. Participants then completed their first health coach visit in person. Participants randomized to receive the I-STAND intervention arm were also provided a Jawbone UP band and trained on how to use it. The baseline visit lasted 1.5 to 2 hours.

Participants also completed an in-person assessment at 3 months post-randomization. Similar to baseline, each person wore an activPAL device for 7 days prior to the visit to assess active and sedentary behavior. During the 3-month visit, the biometric assessments and blood draw were repeated by a blinded study staff member, and a follow-up questionnaire was also administered. Participants received $50 each for completing the baseline and 3-month visit. A subsample of I-STAND participants (n=22) were invited to participate in a separate qualitative exit-interview following study completion. Interviews were conducted by phone within 10 days of the final session. Additional study contacts are outlined as part of the descriptions of the intervention and control conditions (below).

### Randomization & Blinding

Randomization occurred during the in-person baseline visit. The health coach used an automated macro, developed and overseen by the study statistician in Stata, to process the participant’s downloaded baseline activPAL data. The macro computed preliminary estimates of activity metrics such as average daily sitting and standing time. Participants were randomized in a 1:1 allocation to I-STAND or the healthy living control. Randomization was stratified by baseline average daily sitting time (≥9 hours vs <9 hours), in permuted blocks of randomly varying size (2 or 4). Staff responsible for collecting baseline and follow-up data were blinded to participants’ treatment arm. Participants and health coaches were aware of treatment assignment, since individuals received a different intervention depending on their assignment.

### I-STAND Intervention

#### Theoretical framework

The experimental I-STAND intervention was based on relevant behavioral theories including social cognitive theory, the ecological model, and habit formation. Social cognitive theory posits that the interaction of individual, social, and environmental influences impact behavior. Specifically, constructs such as self-efficacy, social support, goal-setting and action planning, and cues were deemed important for inducing changes in sitting behavior. The ecological model specifies the importance of considering influences at the built environment level including the home and neighborhood environment which could shape sitting behaviors [29]. Principles of habit formation suggest that unconscious and automatic processes typically underlie decisions to sit. Bringing these decisions into conscious awareness will help make decisions to stand (instead of sit) more automatic over time [30].

#### Intervention Development

In our prior work, we developed a theory-based ST reduction intervention (using the theories above) and tested it over 8 weeks among older adults with obesity [22]. We then conducted in-depth qualitative interviews to refine and improve the program [27]. The program resulted in a 30-minute reduction in sitting time, comparable to other preliminary studies in older adult populations. The interviews suggested that sitting is a highly ingrained habit often performed unconsciously and additional prompts were suggested to help constantly remind participants to bring their sitting habits into conscious awareness. These findings further informed the design of the I-STAND intervention.

#### Format

I-STAND consisted of 2 in-person health coaching sessions (the first immediately following their baseline measurement visit and the second 1 week later), 4 follow-up health coaching phone calls (every 2 weeks after the first 2 in-person sessions), and written materials. Participants were also offered email reminders to work on their individual goals on the off-weeks of the biweekly calls.

#### Key Components

I-STAND combined the behavioral theories into an approach that focused on using inner, outward, and habit reminder strategies to enhance awareness of sitting behavior and enabled participants to make simple changes that would enhance self-efficacy and reduce sitting time (see Table 1). One of the main tools provided to participants was a Jawbone UP band (Jawbone®, San Francisco, CA) to provide gentle vibrations every 15 minutes of inactivity to remind participants to take breaks from sitting regularly throughout the day (serving as an outward reminder) [31]. In addition to reminder strategies, key components included: 1) a workbook with biweekly content focusing on the various types of reminder strategies, which was used with each health coaching session; 2) feedback charts were provided to participants based on their activPAL wear at baseline and wearing the device at 2 additional check-in points 1 week following the baseline week and at the study mid-point (around week 6). The feedback charts included both numeric and graphic depictions of average daily waking time spent sitting, standing, and stepping, as well as their total breaks from sitting, sitting bouts lasting longer than 30 minutes, and step count; and 3) health coaching sessions as described below. Table 1 provides an overview and descriptions of the I-STAND intervention components.

#### Health Coaching Sessions

Sessions focused on using different types of reminders, building self-efficacy through motivational interviewing strategies, problem-solving barriers, and setting an action plan consisting of graded individualized goals using the workbook which contained action planning and goal-tracking worksheets. At the first in-person intervention visit, health coaches met with participants for 1 hour to develop rapport, learn more about their daily activities, elicit motivations for joining the study, provide an intervention overview, and introduce and review study tools, including the workbook, feedback chart, and Jawbone UP wristband. They also reviewed safety information to ensure that participants would not injure themselves by standing more (eg, stand on a cushioned surface, gradually build the amount of standing time). Health coaches then worked with the participants to set an action plan with obtainable goals, using tailored reminder strategies. During the week following the baseline week, participants wore another activPAL monitoring device and returned in person to meet with the health coach. The second in-person visit, which lasted about 45 minutes, focused on reviewing participant progress on their goals and problem-solving barriers with the assistance of a second feedback chart from wearing the activPAL the prior week; learning about additional reminder strategies; and setting goals for the next 2 weeks.

**Table 1:** Overview of I-STAND Intervention Components.

Component description		Examples of content
Health coaching sessions: 2 in-person and 4 phone calls		Motivational interviewing to identify values and support goal attainment Learning about reminder strategies and selecting personalized reminders to help achieve goals Enhancing self-efficacy for sitting reduction Problem-solving identified barriers to achieving goals Reviewing feedback charts at in-person sessions and at mid-point Action planning including setting stepped goals building towards a 1-hour reduction in sitting time
Feedback charts: Provided 3 times during the intervention		Color graphs and tables showing sitting time, standing time, breaks from sitting, steps, number of sitting bouts lasting longer than 30 minutes Reviewed during health coach sessions at baseline, 1 week, and 6 weeks
Workbook: Provided at first in-person session		Written educational materials Action-planning pages Goal-tracking forms Home environment audit form
**Reminder strategies**		
	Inner: Internal or bodily cues	Using mindfulness to be more aware of how body feels when sitting Standing up anytime you notice your body feeling uncomfortable
	Outward: Cues in the environment	Using the Jawbone UP band, a kitchen timer, or another identified environmental cue Making environmental changes to the home based on audit results (e.g. setting up a standing work space, finding a counter on which to read the newspaper, moving furniture to create room to stand)
	Habit: Ingrained daily habits that can be used as cues	Standing for 5 minutes while engaging in daily habits such as drinking coffee, reading the newspaper, talking on the phone Standing for 5 minutes after doing a daily habit like taking medication or going to the bathroom

Thereafter, health coaches met with participants by phone every 2 weeks (for approximately 20 to 40 minutes for each session) to review progress on goals, problem-solve barriers, use the workbook to guide participants on different types of reminders, and set new action plans at the end of the visit. Additional topics covered in the workbook and health coaching sessions included social support, social environment and norms, conducting a home environment audit, and making home and/or work environment changes based on the audit results.

### Healthy Living Control Condition

Participants in the control condition received 1 in-person health coaching session (after the baseline measurements were completed) followed by 5 mailed contacts. The program was based on usual care that is available to members of KPWA. At the in-person session, participants were provided with a workbook consisting of health education on a variety of topics relevant to aging including depression, advance directives, nutrition, sleep, pain, and bladder control. Participants were instructed to select 1 topic to work on every 2 weeks. Content was derived from online educational information available to KPWA members, which was approved by Kaiser Permanente physicians. During the in-person health coaching session, participants then worked through a goal-setting worksheet with the health coach to help get them oriented to their program. Every 2 weeks, participants received a check-in letter and were asked to complete a form to mail back regarding their progress with their goals.

### Health Coach Training and Fidelity

The I-STAND and Healthy Living conditions were delivered by 2 health coaches who had relevant degrees but no prior experience with health coaching. They were trained by the study principal investigator who is a licensed clinical psychologist (DER) to use motivational interviewing strategies (eg, reflective listening, open-ended questions, affirmations, and summaries) and problem-solving techniques to support behavior change. Fidelity was enhanced by using structured scripts for each session and materials in a study workbook specific to the intervention and control group. Initial sessions were audio-recorded and reviewed to support health coach training. All intervention contacts were tracked in a Microsoft Access tracking database.

### Assessment Measures

The primary outcome was total daily waking hours spent sitting measured by the activPAL micro device (PAL Technologies Ltd, Glasgow, UK). The activPAL was used because it has been feasible in other studies with older adults [22,24,32], is sensitive to change,[22,33] and has high validity in comparison to direct observations [28,34,35]. The device was initialized, sealed in a waterproof casing and then adhered to the front- center thigh with a waterproof medical adhesive (Tegaderm). Participants were instructed not to remove the device but they were given additional materials for affixing the device in the event that the adhesive became compromised or if they developed any irritation. They were provided with logs to track their sleep time each day they wore the device. The data were downloaded and processed using proprietary activPAL software and programs developed for Stata and R statistical software packages. The processing programs removed logged sleep time from the data to calculate waking hours spent sitting. Similar to standard procedures for accelerometer processing, data were considered valid if wear time was greater than 10 hours per day with a minimum of 4 valid days of data for each assessment period [8,36,37]. To account for variations in wear time, activPAL outcomes will be adjusted for wear time. In addition to sitting time, activPAL will be used to assess secondary outcomes including average daily sit-to-stand transitions, standing time, steps, and bouts of sitting longer than 30 minutes.

Other secondary outcomes included physiologic measures and a battery of physical measures thought to be sensitive to changes in ST and relevant for chronic disease. Physical function was measured by the Short Physical Performance Battery, which objectively evaluated lower extremity function with tasks for balance, gait speed, and lower-extremity strength (chair rise) [38,39]. Cardiometabolic outcomes (fasting glucose and a cholesterol panel) were assessed by finger prick using an Alere Cholestech LDX System machine and Lipid + Glucose cassettes. This device has shown very good agreement with established laboratory methods [40-42]. Blood pressure was measured on the left arm using an Omron HEM-907XL digital monitor. Blood pressure was assessed 3 times and the average of the latter 2 measures used.

Exploratory outcomes included cognitive function as measured by the Trail Making Test Parts A and B (to assess psychomotor speed and fluid cognitive abilities) [43,44]. Time to complete each task as a raw score will be used in analyses weight which was measured with a calibrated portable digital scale (Tanita HD-351) and height with a stadiometer (Seca 213). Waist circumference was measured twice at the superior border of the iliac crest. The average of 2 measurements will be used in our analyses [45]. Additional exploratory outcomes were self-reported and included benefits and barriers of sitting reduction [46], self-efficacy for reducing sitting time [46-49], habit formation (Self-Report Habit Index) [50], quality of life with the Patient-Reported Outcomes Measurement Information System global scale [51], and depressive symptoms with the Patient Health Questionnaire-8 [52,53].

### Qualitative Assessment

Qualitative exit-interviews lasted about 45 minutes and followed a semi-structured interview guide. The semi-structured interview guide was intended to capture feedback on the acceptability of the intervention, barriers and facilitators to sitting reduction, and perceived health impacts of sitting reduction. Only I-STAND participants were interviewed. Due to scheduling and other logistics, 22 of the 29 intervention participants were interviewed. The interviews were audio-recorded and transcribed. A formal qualitative analysis using thematic analysis and a group of coders will be undertaken to identify barriers and facilitators to sitting reduction and guide future refinements to the I-STAND intervention.

### Statistical Analysis

The primary outcome will be defined as the change between baseline and 12 weeks in daily sitting time during waking hours, adjusted for wear time. Sitting time adjusted for wear time is a percentage calculated per day as: 100*(sitting time/hours device was worn during waking hours). This measure is averaged across valid wear days within an assessment period. Linear regression models will estimate the difference in mean change in adjusted daily sitting time from baseline to 12 weeks between the healthy living and I-STAND intervention groups. We will adjust for baseline sitting time and important potential confounders. Our primary analysis will include participants with valid sitting time outcome data at both baseline and 3-months data (complete case approach). We will conduct sensitivity analyses including all randomized participants and assuming no change (baseline value carried forward) for participants lost to follow-up. Similar analyses will assess the impact of the intervention at 3 months on secondary outcomes. If linear regression normality assumptions are violated, we will consider transformation of the outcome measures.

#### Power

Based on preliminary data from our prior work [22], we estimated the change from baseline in sitting time adjusted for wear time would have a standard deviation of 8.3%. Assuming an 80% follow-up rate, a sample of 60 (30 in each arm) was estimated to provide 80% power to detect a between-group difference in sitting time adjusted for wear time of about 60 minutes per day.

## Results

**Figure 1:**
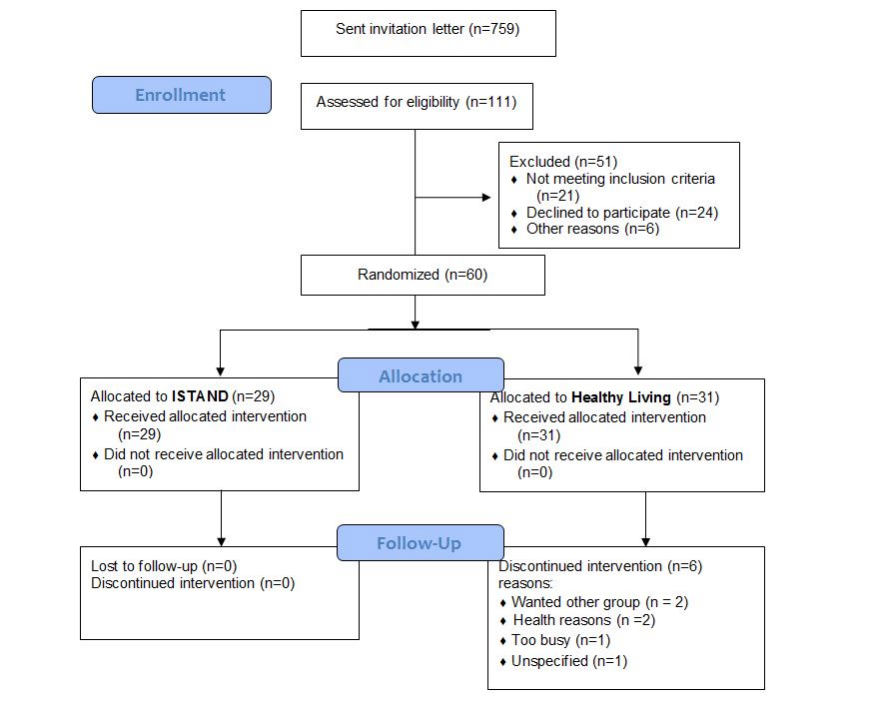
Consolidated Standards of Reporting Trials flow diagram.

Study enrollment was completed. As Figure 1 depicts, 111 (14.6%) of those mailed a study invitation letter responded by calling the study phone line. Of these, 60 were randomized (7.9% recruitment rate). A total of 29 participants received the I-STAND intervention with no drop-outs over 3 months. Thirty-one participants were randomized to the Healthy Living control condition and 6 (19%) dropped out. Data processing and analysis is currently underway.

## Discussion

### Study Rationale

Given the high levels of ST and low physical activity among older populations at risk for chronic conditions, alternative health-promoting approaches are needed. We were able to easily recruit a sample of older adults with a high level of ST to test our intervention. The research base currently lacks evidence from randomized controlled trials to reduce ST among older adults and the current study will be an important contribution to the field. Published trials from adult populations have used standing desks and motivational enhancement to reduce sitting time [19,54]. More recent interventions have incorporated wearable technologies to help provide reminders to take frequent breaks from sitting [55,56]. Only a few studies to date have targeted older adults [21,24-26,56] and the majority were pre-post test studies. While standing desks are effective, [19] they are not as applicable to populations that are largely not working like older adults. Therefore, our approach combined various strategies, including cues from a wearable technology, to remind participants to frequently take breaks from sitting and provide environmental supports for standing. Our findings will elucidate the effectiveness of such approaches.

### Strengths and Limitations

The main limitations of the study included our inability to study outcomes longer than the 12-week study period. Future studies would benefit from longer term follow-up. Another limitation is that there was differential drop-out by condition. The reduced interaction with healthy living participants may have contributed to greater drop-out in this group. Analyzing the study results will help determine the characteristics of completers and non-completers so we can better understand whether our results will be externally valid. Strengths include the use of mixed methods, objective measures of our primary outcomes, inclusion of cardiometabolic outcomes, and the high-risk target population.

### Conclusions

The findings from our study will inform approaches to reducing sitting among a high-risk population. Furthermore, the outcomes will provide novel information on whether sitting reduction results in meaningful changes in health. Data from randomized trials are needed to inform public health guidelines on sedentary behavior.

## Abbreviations

KPWA: Kaiser Permanente Washington
ST: sedentary time

## Appendix

Multimedia Appendix 1 CONSORT-EHEALTH checklist (V 1.6.1).
